# Multiple and Extra-Pair Mating in a Pair-Living Hermaphrodite, the Intertidal Limpet *Siphonaria gigas*

**DOI:** 10.1093/iob/obaa013

**Published:** 2020-04-29

**Authors:** Jessica L B Schaefer, John H Christy, Peter B Marko

**Affiliations:** 1 Department of Biology, University of Hawai‘i at Mānoa, 2538 McCarthy Mall, Edmondson Hall 216, Honolulu, HI 96822, USA; 2 Smithsonian Tropical Research Institute, Balboa, Ancón, Apartado 0843-03092, República de Panamá

## Abstract

Pair-living is a common social system found across animal taxa, and the relationship between pair-living and reproduction varies greatly among species. *Siphonaria gigas*, hermaphroditic pulmonate gastropods, often live in pairs in the rocky intertidal zone of the tropical Eastern Pacific. Combining genetic parentage analysis using four polymorphic microsatellite loci with behavioral observations from a 10-week field study, we provide the first description of the mating system of a *Siphonaria* species incorporating genetic data. *S. gigas* mated both within-pair and extra-pair and three out of four paired *S. gigas* individuals produced egg masses with extra-pair paternity. Multiple paternity was detected, but at a relatively low frequency (19% of egg masses) compared to other marine gastropods. Behavioral data indicate one potential advantage of pair-living: paired *S. gigas* produced almost twice as many egg masses as their solitary counterparts over four reproductive cycles. These observations, together with constraints on the movement of *S. gigas*, suggest that pairing may ensure mate access and increase reproductive success.

## Synopsis

Apareamiento múltiple y extrapareja en un hermafrodita que vive en pareja, la lapa intermareal *Siphonaria gigas* (Multiple and extra-pair mating in a pair-living hermaphrodite, the intertidal limpet *Siphonaria gigas*). Vivir en pareja es un sistema social común que se encuentra en los taxones de animales, y la relación entre la vida en pareja y la reproducción varía mucho entre las especies. *Siphonaria gigas*, gasterópodos pulmonados y hermafroditas, a menudo viven en parejas en la zona rocosa intermareal del Pacífico oriental tropical. Combinando el análisis de parentesco genético utilizando cuatro loci de microsatélites polimórficos con observaciones de comportamiento de un estudio de campo de 10 semanas, proporcionamos la primera descripción del sistema de apareamiento de una especie *Siphonaria* que incorpora datos genéticos. *S. gigas* se apareó tanto dentro como fuera de la pareja, y tres de cada cuatro individuos de *S. gigas* emparejados produjeron masas de huevo con paternidad extrapareja. Se detectó paternidad múltiple, pero a una frecuencia relativamente baja (19% de las masas de huevo) en comparación con otros gasterópodos marinos. Los datos de comportamiento indican una ventaja potencial de la vida en pareja: *S. gigas* emparejado produjo casi el doble de masas de huevo que sus contrapartes solitarias durante cuatro ciclos reproductivos. Estas observaciones, junto con el movimiento restringido de *S. gigas*, sugieren que el emparejamiento puede garantizar el acceso de pareja y aumentar el éxito reproductivo. Translated to Spanish by YE Jimenez (yordano_jimenez@brown.edu)

## Introduction

Animal mating and social systems both influence and are influenced by the spatial distribution of individuals within populations. As sexual reproduction involves the union of gametes from different individuals, spatial proximity is often necessary for animals to mate and reproduce. Pair-living or social monogamy is both a spatial phenomenon and social system in which pairs of conspecifics live together for an extended period of time (Wickler and Seibt 2010). Pair-living is found across a variety of organisms with different modes of reproduction, from snails to primates, and including hermaphrodites and animals with separate sexes (crustaceans: Baeza et al. 2009; Detto and Backwell 2009; polychaetes: Sella and Lorenzi 2000; reef fish: Brandl and Bellwood 2014; mammals: Lukas and Clutton-Brock 2013; birds: Black 1996; Griffith et al. 2008 ).

Numerous studies of vertebrates indicate that pair-living evolves when natural selection favors biparental care, cooperative territoriality, and/or mate guarding (Emlen and Oring 1977; Lukas and Clutton-Brock 2013). However, not all pair-living species have these behavioral traits (e.g., Sella and Lorenzi 2000; Wong and Michiels 2011; Baeza et al. 2016a), suggesting there are other evolutionary drivers of pair-living. Baeza and Thiel (2007) proposed a conceptual model for symbiotic crustaceans, predicting that monogamy may be optimal when hosts are rare and support few individuals and the cost of switching hosts is high (Baeza and Thiel 2007). More broadly stated, pair-living may be favored when refuges are scarce and moving between them is risky (e.g., due to predation), constraining an individual’s ability to find a mate. This idea has found support in studies of symbiotic marine invertebrates (Baeza 2008, 2010; Pfaller et al. 2014), however, it is unknown whether environmental constraints might favor pair-living in free-living organisms. Biparental care, cooperative territoriality, mate-guarding, and Baeza and Thiel’s environmental constraints hypothesis share the assumption that pair-living benefits survival, reproduction, or both. Thus, testing hypotheses for the evolution of pair-living in any organism rely on knowledge of the organism’s ecology, reproductive biology, and mating system.

Pair-living animals may or may not mate with one another, whereas sexual monogamy entails exclusive reproduction between two individuals. Genetic studies of pair-living animals have revealed a range of parentage scenarios, from high frequencies of mixed-paternity or mixed-maternity broods (e.g., mixed-paternity: Griffith et al. 2008 ; Ophir et al. 2008; mixed-maternity: DeWoody et al. 2000) to sexual monogamy (e.g., Piper et al. 1997; Griffith et al. 2008 and references therein). Multiple paternity occurs when a female produces offspring sired by multiple males, which may confer various adaptive benefits. In some cases, females that mate multiply obtain direct benefits though receipt of nuptial gifts, such as nutrient-containing spermatophores, that increase female fecundity or longevity (Fedorka and Mousseau 2002; South and Lewis 2011). When multiple mating results in multiple paternity, females may receive indirect benefits including increased genetic diversity or genetic quality of their offspring (Arnqvist and Nilsson 2000; Griffith et al. 2008; Brouwer et al. 2010; Slatyer et al. 2012 ). Both direct and indirect benefits may drive selection on pair-living animals to mate and fertilize offspring with individuals besides their social partners.

Only one study has described pair-living in a mollusk, *Siphonaria gigas*, an intertidal gastropod that lives in pairs at a rocky intertidal site on the Pacific coast of Panama (Lombardo et al. 2013). *Siphonaria* is a genus of hermaphroditic pulmonate gastropods with internal fertilization, sometimes referred to as “false limpets” (Hodgson 1999), and includes over 40 species found on intertidal shores world-wide (Dayrat et al. 2014). *Siphonaria gigas*, the largest members of *Siphonaria*, are distributed from Mexico to Peru in the tropical Eastern Pacific Ocean (Keen 1971). Individuals of *S. gigas* establish “home scars” by growing their shells to precisely fit the substrate at a fixed location, leaving their home scars for limited periods to graze on encrusting algae and cyanobacteria and to reproduce (Levings and Garrity 1984; Hodgson 1999).

The movement of *S. gigas* is highly constrained spatially and temporally by environmental conditions. *Siphonaria gigas* leave their home scars only while the substrate is wet: when splashed by waves on falling and rising tides during the day and for longer periods at night. They remain on their scars when immersed in water and are rarely seen moving over dry rocks, night or day (Garrity 1984). Homing is critical for survival, as these limpets face greater risk of mortality due to desiccation and predation when off scar (Garrity and Levings 1983). When they do leave their scars, individuals typically move within a meter radius (Levings and Garrity 1984; J. Schaefer et al., personal observations). *Siphonaria gigas* do not self-fertilize, so access to mates is necessary for reproduction, yet restricted movement suggests they infrequently encounter other individuals (Lombardo et al. 2013).

**Fig. 1:**
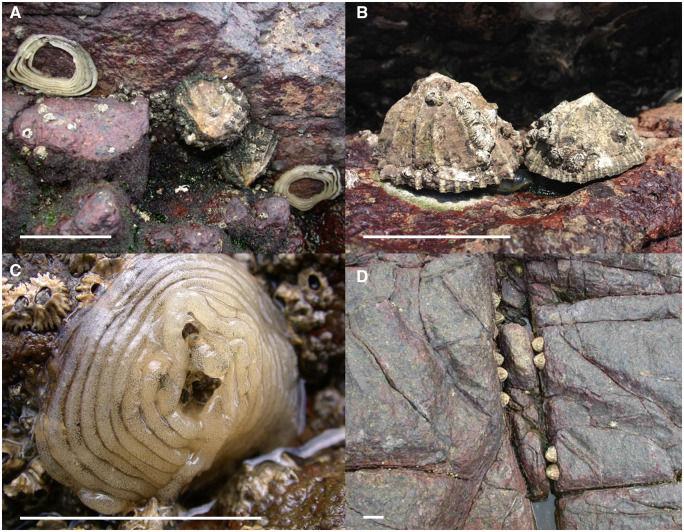
*Siphonaria gigas* and their egg masses at Punta Culebra: (**A**) pair of limpets on home scars and their egg masses; (**B**) limpets facing each other and mating; (**C**) *S. gigas* egg mass; (**D**) paired and solitary limpets on their home scars at Punta Culebra. White bars indicate ∼5 cm. Photographs by J. H. Christy; photo (D) was previously published in Lombardo et al. (2013).

Lombardo et al. (2013) surveyed a population of *S. gigas* at Punta Culebra, Panama and found that 75% of limpets occurred in pairs on adjacent home scars, typically so close that their shells touched when both were on their scars (Fig. 1A;Levings and Garrity 1986; Lombardo et al. 2013). Pairs of *S. gigas* can persist for months (Levings and Garrity 1986) and some pairs have persisted at least 5 years (J. H. Christy, personal observation). While the majority of mature adults live in pairs, the remaining, unpaired limpets are found as solitary individuals or in clusters of several closely spaced but unpaired individuals. Thus, intrapopulation variation in social status provides an opportunity to measure the reproductive consequences of pair-living in a natural setting. *Siphonaria gigas* lack parental care, mate-guarding, and territorial behavior, so alternative factors must lead to pair-living.

In 2016, we revisited this same population at Punta Culebra to gather behavioral, reproductive, and genetic data for this study. The aim of this study was to describe the mating system of *S. gigas* in the context of pair-living and determine the relationship between the spatial distribution and reproduction of *S. gigas*. To achieve this, we combined observations of limpet behavior and reproductive output with genetic parentage analysis of the same limpets and their putative offspring. Specifically, we tested whether pairs of limpets are sexually monogamous and whether multiple paternity occurs within egg masses. This article provides the first description of the mating system of any species of *Siphonaria* utilizing genetic parentage analysis.

## Methods

### Study organism and sampling location

Fieldwork was conducted at Punta Culebra, Panama (8.9119° N, 79.5297° W), in an intertidal area of eroded massive basaltic platforms and a few boulders. A population of *S. gigas* at Punta Culebra was previously surveyed, revealing that individuals preferentially live along horizontal fissures in the rock, with some on exposed horizontal or vertical rock faces and very few inhabiting tide pools or boulders (Levings and Garrity 1984; Lombardo et al. 2013). Each limpet has a home scar, which is recognizable by its lighter coloration due to the absence of the blue-green algal crust that covers most of the substrate. *Siphonaria gigas* exhibit strong reproductive synchrony producing benthic egg masses on the semimonthly neap tides during the rainy season. Each egg mass contains >75,000 embryos on average (Levings and Garrity 1986). Embryos develop in the egg masses for 7–10 days before hatching and entering the plankton.

The “social status” of *S. gigas* individuals can be classified as paired, solitary, or grouped based on their home scar location relative to their neighbors’ home scars. Paired limpets have home scars immediately adjacent to one another; grouped limpets are those living in clusters of three or more; solitary limpets are not directly adjacent to any other limpets when on their scar (Fig. 1). Lombardo et al. (2013) showed that pairing occurs significantly more often than expected by chance, with 75% of *S. gigas* in rock fissures at Punta Culebra living in pairs. The frequency of pairing is also negatively related to density, indicating pair formation does not result from crowding (Lombardo et al. 2013).

In May 2016, we tagged 37 solitary *S. gigas* and 74 individuals in 37 pairs by adhering spots of waterproof epoxy putty (PC Marine) to their shells and writing unique identification numbers on the putty spots. The shell length of each limpet was measured to the nearest millimeter along the anterior-posterior axis, and the distance between solitary limpets and their closest neighbor was measured to the nearest 0.5 cm. Shell lengths of the paired and solitary limpets that were marked were compared using a Wilcoxon rank-sum test.

### Behavioral observations and egg mass production

We visited the study site daily from May 13 to July 21, 2016 (during the rainy season, when *S. gigas* produce egg masses) and monitored the movements, social status, mating behavior, and reproductive output of marked *S. gigas*. Limpets were observed during daytime low tides for 1–4 h daily between 8:00 and 18:00 h, depending on the low tide time. Due to the physical complexity of the habitat and distance between marked limpets, we were unable to observe all marked limpets continuously. Each day we recorded any changes in social status and home scar location of all marked limpets. Mating is indicated when two adjacent limpets raise their shells, touch, overlap, and slowly contract tissues of their anterior bodies where their genital openings are located and remain in this position for several minutes (Fig. 1B). Previous studies at this site indicate that mating occurs predominantly during afternoon low tides several days prior to egg deposition (R. Lombardo and J. H. Christy, unpublished data). We recorded the date and identities of all mating limpets we saw.

We recorded the number of egg masses produced by each marked limpet over four semi-lunar reproductive cycles on May 28, June 13, June 28, and July 12 (±1 day). On these days, we monitored egg production continuously from when limpets were exposed to the air by the falling tide until they were covered again by the rising tide (∼5 h in the afternoon). We noted which individuals produced egg masses and marked the location of each egg mass with an epoxy tag on the rock for later sampling (see “Tissue collection for genetic analysis” section). To allow the embryos to grow larger, egg masses were sampled 6 days after deposition; to protect the masses from fish predation prior to sampling, we installed predator exclosures over each egg mass by affixing wire mesh caps to the rock with waterproof epoxy putty.

To compare total egg mass production between paired and solitary limpets, we limited the dataset to eggs produced by marked limpets that maintained the same social status and home scar throughout all four reproductive cycles (paired: *n* = 56; solitary: *n* = 21). This constraint minimized the potential confounding influence of a limpet’s previous social status on its egg mass production. In addition, *Siphonaria* possesses a spermatheca, an organ for receiving and storing sperm (Pal et al. 2006), so limpets that change home scar locations between reproductive cycles may be able to retain sperm from previous partners. Because the data on egg mass output were non-normal and slightly overdispersed, we used a negative binomial generalized linear model (GLM) with a log link function. The GLM was constructed using the MASS package (Venables and Ripley 2002) in R (version 3.3.1, R Core Team 2016). The dependent variable in the GLM was the total number of egg masses produced over the four cycles, and the model included social status, shell length, and their interaction as factors. The interaction between shell length and social status was not significant and was excluded from the final model.

### Tissue collection for genetic analyses

We collected samples from seven egg masses deposited by paired limpets (referred to as “paired masses”) and nine egg masses deposited by solitary limpets (“solitary masses”) for genetic parentage analysis. Because we saw the individually marked limpet that deposited each egg mass, the maternal parent of each mass was known; for paired limpets, the partner of the maternal limpet was the putative paternal limpet (i.e., putative sire). Five pieces were excised from each egg mass (total <5% of the mass) using a scalpel and forceps. The pieces were taken from positions haphazardly spaced around the spiral-shaped egg mass to account for potential spatial structure of paternity in the mass (i.e., uneven mixing or differential use of sperm). All egg mass samples were collected during the fourth reproductive cycle, from July 17 to 18, so they are temporally comparable.

To sample adult tissue, we removed each limpet from its home scar and non-destructively collected a 1 mm^2^ piece of foot tissue with dissecting scissors. Adult tissue samples were collected from the maternal parents of 14 of 16 sampled egg masses and from the putative paternal parents of 4 of 7 paired egg masses. The remaining maternal and putative paternal limpets were not sampled because they remained clamped on their home scars and could not be removed non-destructively. In some cases, both members of a pair of limpets produced egg masses and we sampled both masses; this is true for four of seven paired masses in the parentage analysis. Thus, the maternal parents for these egg masses are also considered putative sires for the egg masses of their partners. The samples were preserved in 99% ethanol and shipped to the University of Hawai‘i at Mānoa for genetic analysis.

### Deoxyribonucleic acid extraction, microsatellite discovery, and development

Four *S. gigas* adults were selected for microsatellite discovery by constructing shotgun genomic libraries based on a simplified restriction-associated digestion sequencing protocol (after Toonen et al. 2013; Supplementary methods Part 1). The libraries were sequenced on an Illumina MiSeq platform with V3 chemistry and 600 cycles to produce 300 bp paired-end reads; all sequencing was carried out at the University of Hawai‘i Advanced Studies in Genomics, Proteomics, and Bioinformatics. The resulting sequences from each library were trimmed and assembled with SeqMan NGen 12 (DNASTAR, Inc.) with a minimum quality of 30, minimum length of 50 bp, and minimum depth of 10 reads per contig. The assembled sequences were then imported to Geneious version 11.1.4 (http://www.geneious.com/) and trimmed a second time using the BBDuk Geneious plugin (Bushnell, https://sourceforge.net/projects/bbmap/) to remove adapters, with the same minimum quality and length parameters.

To isolate microsatellites in each of the four assembled libraries, we utilized Phobos Tandem Repeat Finder (Mayer 2006, Phobos Geneious plugin). We searched for perfect di-, tri- and tetra-nucleotide motifs with a minimum of six repeat units. The microsatellites were manually screened and a subset of 22 was selected for further development based on number of repeat units, depth of coverage, absence of other repetitive sequences flanking the microsatellite, presence in more than one library, evidence of allelic variation, and ability to design primers flanking the microsatellite. We used Primer3 version 2.3. (Untergasser et al. 2012, Primer3 Geneious plugin) to design locus-specific primers with optimal melting temperature of 60°C, optimal length at 20 bp, and product size from 100 to 300 bp (Supplementary Table S1).

We extracted deoxyribonucleic acid (DNA) from the tissue of 19 adult *S. gigas* (including maternal and putative paternal limpets) using the Qiagen DNeasy Blood and Tissue Kit, following the manufacturer’s protocol but with two separate elutions in 30 μL buffer EB. To obtain embryo DNA, five 2 mm × 1 mm × 1 mm pieces of egg mass from different locations on the same mass were pooled in one tube. Mean embryo density, determined by counting the number of embryos in pieces from 17 egg masses, was 47 embryos/mm^3^ (SD = 24). Thus, each 2 mm^3^ piece of egg mass contained ∼100 embryos, resulting in pooled samples of ∼500 embryos for each egg mass. We used a Qiagen kit to extract DNA from the pooled embryo samples but with several modifications (Supplementary methods Part 2). This yielded final concentrations of embryo and adult DNA ranging from 1 to 15 ng/μL.

### Polymerase chain reaction and amplicon sequencing

Microsatellites were amplified using a two-step amplification protocol: the first polymerase chain reaction (PCR) step utilized locus-specific primers to amplify the locus of interest, and a second PCR step utilized barcode primers to tag individual samples with combinatorial barcodes, following Vartia et al. (2016) (Supplementary methods Part 3). In the first step, microsatellites were amplified individually or in multiplex PCRs containing 2–4 primer pairs. Each pair of locus-specific primers were first tested individually to optimize annealing temperature and number of cycles, and multiplexes were formed by combining primers that amplified under similar cycle conditions (Supplementary Table S2). Following the second PCR step, the barcoded, purified PCR products were pooled into libraries containing approximately equal amounts of amplicons from each individual and locus.

The libraries were prepared for sequencing and Illumina TruSeq adapters were incorporated using the KAPA Hyper Prep Kit. Amplicon libraries were sequenced on an Illumina MiSeq platform with V3 chemistry and 600 cycles to obtain at least 2000 reader per locus per individual. Raw reads from the amplicon libraries were trimmed using BBDuk in Geneious with a minimum quality of 30. This step also removed Illumina adapters and discarded reads <50 bp, as the expected microsatellite-containing amplicons were >100 bp. The trimmed reads were paired by name and merged, then sequences in each library were separated by barcode in Geneious and a fastq file was created for each individual (adult or egg mass).

### Microsatellite genotyping and parentage analysis

We utilized the program MEGASAT 1.0 (Zhan et al. 2017) to demultiplex and process amplicon sequence data from adult limpets and egg masses. MEGASAT was designed to score microsatellite genotypes from multiplexed next generation sequence data. The software applies a series of decision rules to distinguish true microsatellite alleles from PCR artifacts (e.g., amplification stutter) and account for amplification bias among alleles (Zhan et al. 2017). The inputs for MEGASAT are a primer file and a set of fastq files. In this case, each fastq file contained sequences from one adult limpet or one egg mass. The primer file included information used to identify reads containing microsatellite regions of interest: the sequences of locus-specific microsatellite primers, 5′ and 3′ flanking sequences for each locus, and the microsatellite repeat unit. The user can also specify “ratios group” values, which control the stringency of MEGASAT in identifying microsatellite alleles compared to PCR artifacts (Zhan et al. 2017). We used the default ratios group values in MEGASAT.

For adult limpets only, MEGASAT was used to assign genotypes to each individual at the 21 microsatellite loci sequenced. We then utilized CERVUS version 3.0.7 (Kalinowski et al. 2007) to calculate the expected heterozygosity, null allele frequencies, and exclusion probabilities for each locus. Loci were tested for deviations from Hardy Weinberg equilibrium (HWE) using a chi square goodness of fit test in CERVUS with Bonferroni and Yates corrections and a minimum expected allele frequency of one.

Histograms of read length for each individual and locus were generated using MEGASAT. We manually checked the genotype of each adult limpet by comparing the pattern of peaks in its read length histogram to the MEGASAT-assigned genotype for that limpet. A diploid individual cannot have more than two alleles per locus; however, the read length histograms for some loci contained numerous peaks of similar size attributed to stutter, making it difficult to determine which read lengths corresponded to true alleles and which were PCR artifacts. Loci with complex peak patterns in the read length histograms were excluded from the parentage analysis due to ambiguity in scoring genotypes. Out of 21 microsatellite loci sequenced, 4 were chosen for use in the parentage analysis because they met all of the following conditions: polyallelic, consistent with HWE, and unambiguous to score.

Focusing on these four loci, we used MEGASAT to assign genotypes and generate read length histograms for the egg mass samples. MEGASAT will call a maximum of two alleles per locus per sample. However, each egg mass sample contained DNA from a pool of ∼500 embryos, so their collective genotype may consist of four alleles per locus (two maternal and two paternal), or more than four alleles if multiple sires fertilized offspring in the same egg mass. To account for this, we examined the read length histograms of egg mass samples and manually corrected MEGASAT-assigned genotypes when there were more than two alleles present. A conservative approach was taken and egg masses were only scored for alleles that were present in at least one adult in the study.

We compared the genotype of each egg mass to its known maternal parent and, for paired masses, the putative paternal parent. A minimum number of sires was calculated for each egg mass at each locus by dividing the number of non-maternal alleles present in the mass by two (i.e., assuming all sires were heterozygous) and rounding up to the nearest integer. We also took a less conservative approach, assuming all sires were homozygous and estimating the number of sires as equal to the number of non-maternal alleles present in the egg mass.

Paired egg masses were categorized as either consistent with genetic monogamy (if all alleles present in the egg mass were found in the maternal and/or putative paternal parent) or not consistent with monogamy (egg mass contained at least one allele found in neither the maternal nor the putative paternal parent). The latter case provides evidence for extra-pair mating.

### Probability of detecting multiple paternity

We used a model developed by Neff and Pitcher (2002) to assess the power of our panel of markers to detect multiple paternity in *S. gigas* egg masses. The probability of detecting multiple (PrDM) mating was calculated for masses sired by two or four limpets and with a range of paternal skew scenarios, assuming in each case that 100 embryos (the maximum allowed by the software) were sampled and genotyped.

### Ethical approval 

This research was conducted under scientific permit SE/A-67-16 and export permit SEX/A-59-16 from the Republic of Panama. All applicable international, national, and institutional guidelines for ethical research were followed.

## Results

**Fig. 2:**
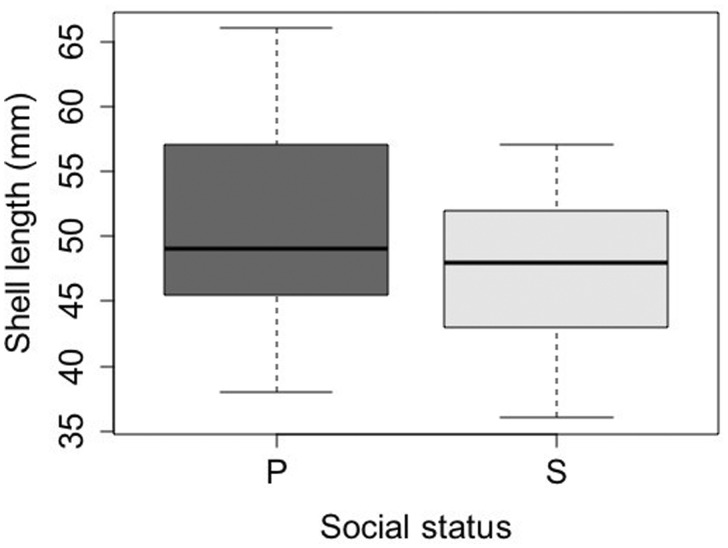
Shell length of paired (*n* = 21) and solitary (*n* = 56) *S. gigas* from which behavior and egg mass data were collected. The box plot shows the median shell length (dark horizontal line), first and third quartiles (box), and minimum and maximum shell length (whiskers) for paired (P) and solitary (S) limpets, respectively.

A total of 37 solitary and 74 paired *S. gigas* (i.e., 37 pairs) were individually marked at the beginning of the study. The distance between solitary limpets and their nearest neighbor ranged from 2.5 to 140.5 cm (median 25.3 cm). The two social classes were similar in shell length (Wilcoxon rank-sum test, *z* = −1.76, *P* = 0.0777), with mean shell length of 50.6 (SD = 6.9) mm in paired and 47.2 (SD = 6.0) mm in solitary limpets (Fig. 2). Three solitary and two paired limpets were lost before the end of the study period, so data on their behavior was gathered for <10 weeks.

### Behavior and social status

**Fig. 3:**
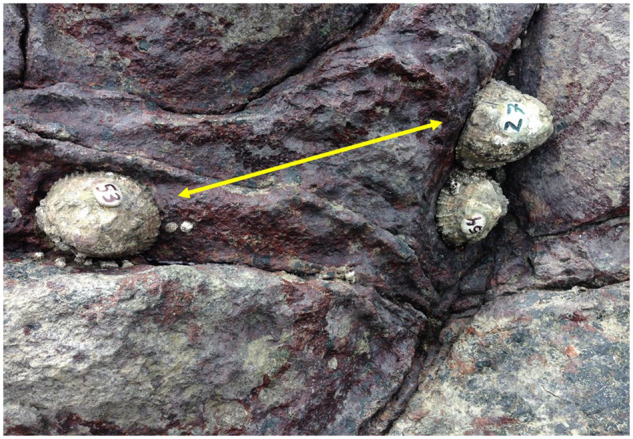
Change in social status involving a pair and a nearby solitary limpet. The limpets marked 53 and 54 were initially paired, and Limpet 27 was initially solitary. This photo shows all limpets sitting on their new home scars after Limpets 27 and 53 switched locations as indicated by the arrow. Photograph by J. Schaefer.

Almost one third (34 of 111) of initially paired and solitary limpets changed social status over 10 weeks of observation. The remaining limpets (21 of 37 solitary and 56 of 74 paired) maintained their original social status and home scar location. Almost half (15 of 37) of solitary limpets became paired by the end of the study: 12 joined another solitary or unmarked limpet to form a new pair, while three solitary limpets replaced one member each from three existing pairs (Fig. 3). Four solitary limpets became part of a group of three or more limpets.

Paired limpets tended to remain in pairs. Only 2 of 37 pairs separated with both members becoming solitary. In one case, the leaving partner established a new home scar 250 cm away from its former partner (which remained in the old location), and in the second case the leaving limpet moved 30 cm away from its former partner. Two paired limpets from separate pairs became solitary when their partners left and established home scars near different limpets. Three additional paired individuals became solitary when each was replaced by another limpet; two of these newly solitary limpets were found inhabiting the old scar of the solitary individual that replaced them (Fig. 3). Five pairs of limpets became trios when a third limpet established a new home scar adjacent to the paired limpets’ existing scars.

We observed *S. gigas* individuals mating both with their social partner (within-pair) and with limpets that were not their social partner (extra-pair). Over 10 weeks, 11 pairs of limpets were observed mating within-pair, and 3 of 11 were observed mating twice within-pair with the second mating event occurring 3, 13, and 18 days after the first. We also observed seven extra-pair mating events, including four instances where two limpets from different pairs mated with each other. Solitary limpets were observed mating with other solitary limpets (*n* = 2), a paired limpet (*n* = 1), and unmarked limpets (*n* = 5).

### Egg mass production

Egg mass production varied between individuals, across reproductive cycles, and by social status. On a single reproductive cycle, individuals produced from zero to two egg masses. Over four reproductive cycles, total egg mass production ranged from zero to five masses per individual.

**Fig. 4:**
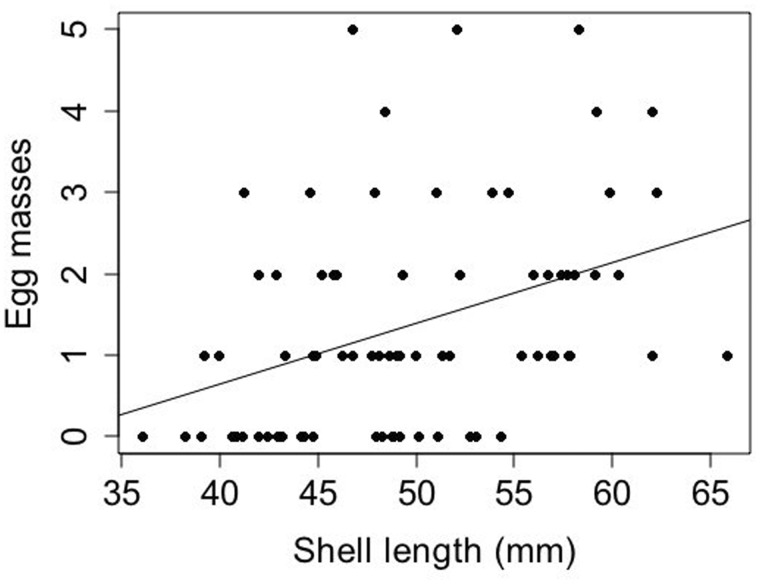
Total egg mass production over four reproductive cycles plotted against shell length of paired and solitary *S. gigas*. There was a significant, but weak positive association between length and the number of egg masses produced (GLM, *z* = 3.10, *P* = 0.0019). Points are jittered for clarity.

**Fig. 5:**
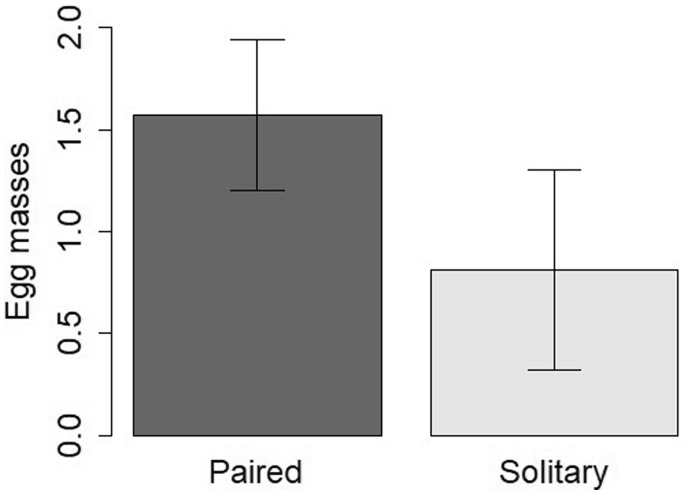
Mean number of egg masses produced by paired (*n* = 56) and solitary (*n* = 21) *S. gigas* over four reproductive cycles. Error bars indicate 95% confidence intervals.

Egg mass output was related to limpet shell length (Fig. 4) as well as social status (Fig. 5). When tested with a GLM, the covariate shell length was significant (*z* = 3.10, *P* = 0.0019, β = 0.0471, 95% CI [0.0173, 0.0772]), although it explained only 14.2% of variation in egg mass production. The relationship between social status and egg mass production was nonsignificant at α = 0.05 (*z* = −1.77, *P* = 0.0762, β = −0.494, 95% CI [−1.070, 0.0279]). However, paired limpets produced almost twice as many egg masses as solitary limpets: the mean total egg mass output over four cycles was 1.57 (SD = 1.37) and 0.81 (SD = 1.08) for paired and solitary limpets, respectively (Fig. 5).

### Microsatellite discovery and genetic analysis

Sequencing of the four genomic libraries yielded from 204,558 to 1,683,261 reads (a total of 3,384,326 reads). Out of the 22 candidate microsatellite loci identified from these libraries, PCR was successful for 21 loci and one locus failed to amplify.

Adult limpets (*n* = 19) and their egg masses (*n* = 16) were genotyped at four polymorphic microsatellite loci that met our minimum criteria for inclusion in the parentage analysis (Table 1). Each locus contained four to seven alleles of different length, and sequence data provided information on the cause of allelic variation at these loci. Nearly all length variation was due to differences in the number of microsatellite repeat units. Along with number of repeat units, locus MS-34 contained a poly-A region within the flanking region that contributed to allele length variation. Two alleles contained nucleotide substitutions, but because each substitution was always associated with a certain unique number of repeats, these nucleotide polymorphisms did not create any hidden allelic variation.

**Table 1:** Summary statistics for microsatellite loci used in the parentage analysis.

Locus	Repeat	*N*_a_	*N*	*H*_o_	*H*_e_	HW	*F*_null_	*P*_excl_
MS-03	AC	5	19	0.684	0.724	>0.05	0.0222	0.292
MS-26	TAA	7	19	0.895	0.856	>0.05	0.033	0.494
MS-31	TC	7	18	0.556	0.603	>0.05	0.0269	0.202
MS-34	TTG	4	19	0.526	0.587	>0.05	0.0276	0.175

Locus name, repeat unit, number of alleles (*N*_a_), number of adults genotyped at that locus (*N*), observed heterozygosity (*H*_o_), expected heterozygosity (*H*_e_), probability of deviation from HWE (HW), estimated null allele frequency (*F*_null_), and exclusion probability (*P*_excl_). The exclusion probability is the probability of excluding a random unrelated individual as parent for a given offspring based on their genotypes at that locus. HWE was tested using a chi square goodness of fit test in CERVUS with a minimum expected allele frequency of one.

Allele frequencies in the adults confirmed that the four loci were suitable for parentage analysis. The loci did not deviate significantly from HWE (*P* > 0.05) and none showed evidence of null alleles, with estimated null allele frequencies <0.04. The number of alleles per locus ranged from four to seven and the mean expected heterozygosity was 0.691 across the four loci. The mean expected homozygosity was 0.307 and the probability of two individuals being homozygous at one locus was 0.307^2^ = 0.0942. The combined probability of exclusion for this set of four loci, that is, the probability of excluding a random unrelated individual as a parent for a given offspring when neither parent is known, based on their multilocus genotypes, was 0.764. With one parent known, the combined probability of exclusion for a second unrelated individual was 0.926.

Genetic analysis of egg mass samples identified between one and five alleles per locus per mass. The egg masses contained at least one maternal allele at every locus genotyped. Genotype data could not be obtained for one locus each for three egg masses due to low read depth, so parentage analysis of those masses was based on just three loci.

### Parentage analysis

Three out of 16 egg masses produced by both paired and solitary limpets showed evidence of multiple paternity, with offspring alleles indicating a minimum of two sires (Table 2). For all three cases of multiple paternity, the maternal limpet was solitary. The remaining 13 of 16 egg masses were consistent with single paternity, since none contained more than two non-maternal alleles at a given locus. Applying the less conservative approach of assuming all sires were homozygous, the number of egg masses with evidence of multiple paternity at one or more loci increased from three to eight egg masses, and the number of sires per egg mass ranged from one to four instead of one to two.

**Table 2:** Results from the test for multiple paternity in egg masses produced by paired and solitary limpets: alleles present in the pooled offspring from egg masses (O), genotype of the maternal limpet (M), and social status of the maternal limpet: paired (P) or solitary (S)

	Maternal	MS-03		MS-26		MS-31		MS-34		
Egg mass	social status	O	M	O	M	O	M	O	M	Min # sires
20	P	113 **117** 119	113 119	**283** 287 295	287 295	57 **59** **61**	57 57	235 238 **244**	235 238	1
21	P	115 **117** 121	115 121	275 281 **295**	275 281	57 59	57 59	238	238 238	1
23	P	115 117 121	NS	275 281 295	NS	57 59	NS	238	NS	1
25	S	**115** 117 121	117 121	272 **277** **281** 283	272 283	57 **61**	57 57	235 238	235 238	1
26	S	115 119	115 119	**275** 287	281 287	**53** 57 **59** **61** **63**	57 57	**234** 238 **244**	232 238	2
27	P	115 117	115 117	**275** 277 281	277 281	**43** 57 61	57 61	235 238	235 238	1
28	S	**113** 115 **119**	115 117	275 277 **283** **287**	275 277	57 **61** **63**	57 57	**232** 238	238 238	1
29	S	115	113 115	ND	275 287	57	57 57	**232** 235 238	235 238	1
30	P	**115** 117	117 117	275 **281**	275 275	57 **59**	43 57	**232** 238	238 238	1
31	P	117	117 117	**275** 287	283 287	**57** 59	59 61	**235** 238 244	238 244	1
32	S	**115** 117 **119**	117 117	**269** **275** **283** 287	287 295	ND	51 51	**232** **235** 238 **244**	238 238	2
33	P	115 **117**	115 115	**275** 277 **287** 295	277 295	57	57 57	**235** 238	238 238	1
34	S	**113** 115	115 115	**277** 281 283	281 283	41 **53** 57	41 57	**232** 235 238	235 238	1
35	S	115 **117** **121**	113 115	275 **281** 287	275 287	57 59	57 59	235 238	235 238	1
36	S	115 117	NS	277 281 283	NS	57 59	NS	232 238	NS	1
37	S	ND	117 119	**275** 277 **281** 283	277 283	57 **59**	51 57	**232** **235** 238 **244**	238 238	2

The maternal genotype is known for these masses, except for two masses for which the maternal parent was not sampled (NS). No data (ND) indicates an individual could not be genotyped at that particular locus. Bold font indicates alleles present in the egg mass that were not found in the maternal limpet for that mass. The minimum number of sires was determined by counting the number of non-maternal alleles present in the offspring, dividing by two, and rounding up to the nearest integer.

Considering the four paired egg masses for which the genotypes of both the maternal and putative paternal limpet were known, we found evidence of extra-pair paternity in three masses (Table 3). Only one egg mass was consistent with sexual monogamy, meaning that all identified offspring alleles in that mass were found in either the maternal limpet or its social partner. The other three egg masses contained alleles at two or more loci that were present in neither the maternal nor putative paternal limpet, indicating extra-pair paternity. All three of these egg masses contained one to two non-maternal alleles at each locus including at least one allele not present in the putative father; thus, they were compatible with a single extra-pair sire (assuming sires were heterozygous, the most conservative approach). Two out of three of the masses with extra-pair paternity lacked alleles from the social partner at one or more loci, excluding this individual as a sire. The alleles present in the third egg mass were compatible with either one extra-pair sire or with two sires: the social partner of the maternal limpet and one extra-pair sire.

**Table 3:** Results from the test for monogamy in egg masses produced by paired limpets: alleles present in the pooled offspring from egg masses (O) and genotypes of their respective maternal (M) and putative paternal (PP) limpets

	MS-03			MS-26			MS-31			MS-34			
Egg mass	O	M	PP	O	M	PP	O	M	PP	O	M	PP	Parentage
20	113 **117** 119	113 119	115 115	**283** 287 295	287 295	272 277	57 **59** **61**	57 57	57 57	235 238 **244**	235 238	238 238	EP
27	115 117	115 117	117 117	275 277 281	277 281	275 275	43 57 61	57 61	43 57	235 238	235 238	238 238	MO
30	115 117	117 117	115 117	275 281	275 275	277 281	57 **59**	43 57	57 61	**232** 238	238 238	235 238	EP
33	115 117	115 115	115 117	**275** 277 **287** 295	277 295	281 283	57	57 57	57	**235** 238	238 238	232 238	EP

The putative paternal limpet was the social partner of the maternal limpet. Parentage was classified as extra-pair (EP) when there was evidence of at least one EP sire, or as consistent with sexual monogamy (MO). Bold font indicates alleles present in the egg mass that were not found in either the maternal or putative paternal limpet for that mass, indicating extra-pair paternity.

### Probability to detect multiple paternity

The power to detect multiple paternity with this set of four loci was high for a range of paternity scenarios (Table 4). With at least 100 offspring genotyped, the PrDM paternity was 94% even when paternity was highly skewed between two males siring 95% and 5% of offspring, respectively. PrDM paternity decreased only when the second sire’s contribution was very small (Table 4).

**Table 4:** PrDM paternity for different numbers of sires and levels of paternity skew (e.g., skew of 50:50 indicates two males each sired 50% of offspring in an egg mass)

Number of sires	Paternal skew (% offspring sired)	PrDM paternity
2	50:50	0.970
2	80:20	0.970
2	95:5	0.940
2	99:1	0.527
4	25:25:25:25	>0.999
4	90:3.3:3.3:3.3	0.999

Each simulation assumes 100 offspring were genotyped.

## Discussion

Genetic data revealed that *S. gigas* can be polygamous and produce egg masses sired by more than one individual. That said, multiple paternity was detected in only 19% (3 of 16) of egg masses, a relatively low frequency compared to other marine gastropods (Angeloni et al. 2003; Dupont et al. 2006; Walker et al. 2007; Brante et al. 2011; Xue et al. 2014; Morales et al. 2016). For example, multiple paternity was found in 89.5% of broods in a muricid (Xue et al. 2014), and Kamel and Grosberg (2012) detected multiple paternity in 100% of *Solenosteira* broods. The number of sires contributing to a single clutch (i.e., the degree of multiple paternity) ranged from two to eight in marine gastropods (Walker et al. 2007; Xue et al. 2014; Morales et al. 2016). In contrast, there was no genetic evidence of more than two sires per egg mass in *S. gigas* when the same approach of estimating minimum number of sires was applied. When a less conservative method was used to calculate the number of sires, assuming all sires were homozygous, the frequency of multiple paternity in *S. gigas* increased from 19% to 50% (8 of 16 masses) and the number of sires ranged from one to four. However, the low probability of two individuals being homozygous at one locus (0.0942) makes the latter estimates less realistic, especially considering information from four loci was used to determine the number of sires. In either case, compared to other marine gastropods with more promiscuous mating systems, the number of sires contributing to *S. gigas* egg masses at Punta Culebra was relatively low.

While the majority of *S. gigas* egg masses were compatible with single paternity, that conclusion depends on the power of the genetic markers to detect more than one sire. Considering several hundred embryos were sampled and pooled from each egg mass, our panel of microsatellites provided a high PrDM paternity of ≥0.94 even assuming paternity was highly skewed at 95% and 5% between two males. This level of paternity skew would be more extreme than typical levels reported in marine gastropods such as knobbed whelks (Walker et al. 2007) and slipper shells (up to five males siring 10.7–46.3% of offspring each, Brante et al. 2011). Thus, the power of our analysis was sufficient to detect multiple paternity in biologically realistic scenarios, strengthening the finding that multiple paternity occurs at a relatively low frequency in this population of *S. gigas*.

Multiple mating is common among hermaphroditic gastropods (Nakadera and Koene 2013) and among pulmonates (Jordaens et al. 2007) and appears to be the norm rather than an exception. The discovery of multiple paternity in *S. gigas* conforms to these patterns, yet the low frequency and degree of multiple paternity distinguish *S. gigas* from other gastropods. We note that while single paternity is suggestive of single mating, single paternity can also follow multiple mating if sperm from just one mating partner is used to fertilize eggs; this could occur as a result of post-mating female choice, last-male sperm precedence, or sperm competition. Proposed benefits of multiple mating include direct, non-genetic benefits to females of receiving male ejaculate (Wagner et al. 2007), indirect benefits such as greater genetic diversity among offspring (Brouwer et al. 2010), facilitation of female choice (for single paternity preceded by multiple mating), and, for males, insurance against predation of inseminated females (Arnqvist and Nilsson 2000). On the other hand, sperm competition among males increases with group size and the rate of multiple mating (Charnov 1982; Tan et al. 2004). The mating system of *S. gigas* is likely influenced by most if not all of these factors, and the most adaptive reproductive strategy must balance male and female fitness.

Genetic results confirmed that social partners in *S. gigas* are not sexually monogamous: three out of four egg masses, for which both the maternal genotype and that of the putative sire were known, displayed evidence of extra-pair paternity at two or more loci. Although the sample size was small, the frequency of extra-pair paternity (75%) was supported by direct observations of extra-pair mating.

Paired limpets in the field mated both within-pair and extra-pair, albeit more often within-pair (17 times versus 7 times). These results are consistent with previous work on *S. gigas* (Levings and Garrity 1986), in which 69% of mating events occurred between limpets who were nearest neighbors (Levings and Garrity did not differentiate between solitary and paired limpets). Tracking the movements and mating behavior of marked paired and solitary individuals demonstrated that limpets living in pairs also mated with limpets who were not their social partner and nearest neighbor. Although we did not make nighttime observations, in a previous, unpublished study (17 observations of 22 individuals over six nights) no limpets were observed mating or depositing egg masses at night (R. Lombardo and J. H. Christy, unpublished data). These results suggest that *S. gigas* usually mate and deposit egg masses during daylight. Furthermore, limpets’ nocturnal movements remained spatially restricted to within 0.75 m of their home scars (R. Lombardo and J. H. Christy, unpublished data), further corroborating that encounters with other limpets occur mainly between individuals whose scars are in close proximity.

Patterns of mating can be asymmetric between members of a social pair. For example, one pair of limpets was observed mating with each other twice—once 13 days before depositing their egg masses and again 5 days after. Parentage analysis of the egg masses produced by each pair member revealed that one limpet’s egg mass was sired within-pair, while the other’s mass contained alleles from an extra-pair sire, indicating the second pair member had mated with another limpet in addition to its social partner. Thus, one limpet received sperm from its partner, while the other either did not receive or did not utilize sperm from its partner to fertilize its eggs. These observations suggest that copulation is not reciprocal in *S. gigas*, even between social partners, consistent with the finding of non-reciprocal copulation in *S. capensis* (Pal et al. 2006).

Social monogamy does not entail sexual monogamy in other taxa, suggesting that pair-living may be beneficial despite ubiquitous extra-pair mating (Fietz et al. 2000; Griffith et al. 2008; Ophir et al. 2008). As in these taxa, selection favoring pair-living in *S. gigas* does not rely on exclusive production of offspring between social partners. The finding of multiple mating in solitary limpets, along with the behavioral and genetic evidence of extra-pair mating in paired limpets, seemingly contradicts the notion that pairing is a strategy to alleviate limited mate access. However, pair-living may still be beneficial if encounters with potential mates are sporadic and limited. *Siphonaria gigas* may mate opportunistically with individuals they encounter while foraging off scar. However, since activity is restricted and limpets spend most of their time on scar, establishing a home scar near a partner could ensure that limpets always have access to a mate, including times when they fail to encounter other limpets while foraging.

Over four reproductive cycles, we found that paired limpets produced more egg masses than solitary limpets, consistent with the idea that one benefit to pair-living is greater female reproductive success. While this relationship was marginally nonsignificant, the difference was substantial, with nearly two-fold greater egg mass output in paired limpets. It is possible that solitary limpets produce fewer, but larger egg masses containing more embryos, or that offspring survival differs by social status. However, the proportion of viable embryos in paired and solitary egg masses did not differ after 8 days of development (J. Schaefer, unpublished data), which is when embryos begin to hatch from egg masses and enter a planktonic phase.

The observation that paired *S. gigas* tended to produce more egg masses than solitary *S. gigas* is consistent with the environmental constraints hypothesis for social monogamy (Baeza and Thiel 2007). Previous studies have established a link between a symbiotic lifestyle, constraints on mate access, and social monogamy in other tropical marine invertebrates; in these organisms, a high risk of predation outside of the host is thought to limit movement and favor social monogamy (Baeza 2008, 2010; Pfaller et al. 2014). Although *S. gigas* is not symbiotic, individuals occupy specific home scars in the intertidal that provide refuge from predation and dislodgement by waves, analogous to the protective function of the hosts of symbiotic marine invertebrates. Since *S. gigas* cannot self-fertilize, they must receive sperm from other individuals to produce fertilized egg masses. Therefore, given their limited periods of movement and the importance of homing (Garrity and Levings 1983; Garrity 1984), *S. gigas* pairing may be beneficial if establishing a home scar directly adjacent to a partner facilitates mate access.

Status switches from solitary to paired occurred more often than paired to solitary transitions, further suggesting a preference for pair-living. Over the 10-week study, 40% (15 of 37) of solitary limpets became paired and only 12% (9 of 74) of paired limpets became solitary; those that did become solitary were often replaced by a different limpet. In these cases, we could not determine whether the incoming limpet inhabited the home scar of the replaced limpet first, preventing the replaced limpet from returning to its scar, or whether the replaced limpet intentionally moved away, leaving its old scar vacant. The first scenario would indicate there is competition for partners, while both scenarios suggest there may be benefits to occupying the previous home scar of another limpet.

The social dynamics observed at Punta Culebra suggest that the relative benefits of living in pairs versus being solitary could vary seasonally or over the lifetime of *S. gigas* individuals. For example, *S. gigas* activity patterns and behavior may be different in the rainy season (May–December) than in the dry season (January–April), since *S. gigas* typically move only when the substrate is wet at low tide (Garrity 1984). Additionally, a reduction in upwelling-driven nutrient availability in the Gulf of Panama during the rainy season (D’Croz and O’Dea 2007) has been correlated with lower cover of benthic algae in the intertidal (A. Sellers, personal communication). Thus, limited resource abundance may place energetic constraints on *S. gigas* movement during the rainy season. Lombardo et al. (2013) showed that 75% of limpets at Punta Culebra were paired in June–July 2004, while we observed a trend of solitary individuals moving into pairs from May to June 2016. Together, these studies point to the value of living in pairs during the early- to mid-rainy season, when benthic algae cover is sparse and *S. gigas* is reproductively active.

If mate access is a key driver of pair-living, the question remains as to why *S. gigas* are not more commonly found in larger groups. One possibility is that competition becomes more intense when more than two limpets form home scars and graze in close proximity. Given their large body size and the low biomass of cyanobacterial crust on which they feed, it seems likely that *S. gigas* are resource limited. This could be tested by examining variation in group size within or among populations with respect to habitat quality/algal abundance. In concordance with this idea, variation in the social organization of other marine invertebrates has been linked to habitat variation (Baeza et al. 2016b). Furthermore, seasonal upwelling and variation in intertidal productivity could drive resource availability for *S. gigas*, influencing the relative value of different social strategies. Long-term tracking of marked individuals would shed light on temporal patterns in social organization with respect to seasonal abiotic factors.

## Conclusion

Behavioral observations of mating combined with genetic parentage analysis indicated that *S. gigas* are not sexually monogamous, including those that live in pairs. Extra-pair paternity and multiple paternity occurred within a single reproductive cycle. The trend toward greater egg mass output of paired limpets, combined with the fact that most limpets at Punta Culebra live in pairs, suggests that pairing confers reproductive benefits. Hermaphroditic animals gain fitness through both male and female reproductive functions, and the influence of social status on male reproductive success of *S. gigas* is unknown. To test for adaptive benefits of pair-living, future studies should measure total fitness, including both male and female reproduction and in terms of the number of offspring produced. Future work should also incorporate genetic sampling of offspring over multiple reproductive cycles to examine temporal patterns in mating and parentage. In addition, studies manipulating the social status of individuals in the field and then monitoring their behavior and reproduction could further elucidate the fitness consequences of pairing in this hermaphroditic gastropod.

## Data accessibility

Genetic datasets analyzed in this study are available in the Dryad repository at https://datadryad.org/stash/landing/show?big=showme&id=doi%3A10.25338%2FB8H038; behavioral and reproductive data are available from the corresponding author upon request.

## Supplementary Material

obaa013_Supplementary_Data
