# Supplementary material for: Multiple and Extra-Pair Mating in a Pair-Living Hermaphrodite, the Intertidal Limpet Siphonaria gigas
Source: Integr Org Biol. 2020 Apr 29;2(1):obaa013. doi: 10.1093/iob/obaa013 (PMC7671124; doi:10.1093/iob/obaa013)
Supplement: obaa013_Supplementary_Data [file obaa013_supplementary_data.docx]

**Supplementary Methods**

*Part 1: DNA extraction and RADseq library preparation*

Four *Siphonaria gigas* adults were selected for microsatellite discovery by constructing shotgun genomic libraries based on a simplified restriction-associated digestion sequencing (RADseq) protocol (after Toonen et al. 2013). First, we extracted genomic DNA from the foot tissue of each individual using the Qiagen DNeasy Blood and Tissue Kit, following the manufacturer’s protocol but collecting three separate elutions of 35, 50, and 50 μl, respectively, from the same spin column using pre-warmed 70°C water. DNA from each elution, which tend to differ in quantity and quality, was quantified using a Qubit® Fluorometer and Qubit® dsDNA HS Assay Kit (Invitrogen, ThermoFisher Scientific). For each individual, elutions were pooled starting with the first (35 μl) elution and adding the second and third elutions successively to obtain a total of at least 1 μg DNA; the pooled elutions were then concentrated by rotary evaporation. Next, DNA from the four limpets was digested in separate reactions containing 272–471 ng DNA, 1 μl DpnII, 5 μl NEBuffer^TM^ 3.1 (New England Biolabs), and water to a total volume of 50 μl. The samples were incubated at 37°C for 3 hours followed by 20 minutes at 65°C, then purified with AMPure XP beads (Beckman Coulter). Four genomic libraries were prepared with the digested DNA from each of the four individuals using the KAPA Hyper Prep Kit (Kapa Biosystems Ltd.) and Illumina TruSeq adapters (Illumina Inc.).

*Part 2: DNA extraction from S. gigas embryos*

Each pooled embryo sample contained approximately 500 embryos encased in individual egg capsules within a gelatinous matrix. We used a Qiagen DNeasy Blood & Tissue kit to extract DNA from the pooled embryo samples but with the following modifications: first, to break up the gelatinous matrix, 540 μl buffer ATL and 0.1 g 0.1 mm zirconia/silica beads were added to each tube and the samples were agitated by shaking for 5 minutes at 25 Hz, then incubated for 10 minutes at 56°C. The agitation and incubation steps were repeated, followed by addition of 60 μl proteinase K and incubation at 56°C for approximately 12 hours. The rest of the extraction followed the Qiagen kit protocol as written but with two separate elutions of 30 μl in buffer EB.

*Part 3: PCR and amplicon sequencing*

Microsatellites were amplified using a two-step amplification protocol: the first polymerase chain reaction (PCR) step utilized locus-specific primers to amplify the locus of interest, and a second PCR step utilized barcode primers to tag individual samples with combinatorial barcodes, following Vartia et al. (2016). A 5’ adapter sequence was added to each locus-specific primer to facilitate binding of barcode primers in the second PCR step. The barcode primers consisted of the complement to an adapter sequence and one of twelve barcode sequences. Forward primers were adapted with Hill, M13, or Neo and reverse primers were adapted with CAG (sequences provided in Vartia et al. 2016). We tested the locus-specific primers with 5’ extensions for secondary structure formation using the IDT Oligo Analyzer tool (<http://eu.idtdna.com/calc/analyzer>) and ThermoFisher Multiple Primer Analyzer (<https://www.thermofisher.com>), and we designed primers to minimize formation of hairpins, primer dimers, and hetero-dimers. Four forward and eight reverse 10 bp barcode sequences were utilized for a total of 32 unique combinatorial barcodes, which enabled us to pool samples prior to sequencing and demultiplex the sequences based on their forward-reverse barcode combination. Some combinatorial barcodes assigned to adult limpets were repeated in the embryo samples; however, amplicons from adults and embryos were always pooled in separate libraries for sequencing, so the samples could be distinguished even though they shared the same barcode sequences.

Microsatellites were amplified individually or in multiplex PCRs containing 2–4 primer pairs. Each pair of locus-specific primers were first tested individually to optimize annealing temperature and number of cycles, and multiplexes were formed by combining primers that amplified under similar cycle conditions. Each 10 μl multiplex reaction consisted of 0.5 μl template DNA, 0.2 μmol each forward and reverse primer, and 5 μl MyTaq Red Mix (Bioline). In addition, 0.5 μl DMSO and 0.5 μl BSA were added to PCR reactions for embryo samples (except for locus MS-26, which did not include the additives and utilized 0.5 μl of 1:10 diluted embryo DNA as the template). Annealing temperatures (T_a_) for the multiplex PCRs ranged from 55–60°C depending on the primers; thermocycling profiles consisted of a 90 s initial denaturation step at 94°C, followed by 25–30 cycles of 20 s at 94°C, 20 s at T_a_, 25 s at 72°C, and a final extension for 10 minutes at 72°C (Supplementary Table 2).

After the first PCR, products were individually purified either by bead cleaning or with the Qiagen PCR purification kit. Bead cleans utilized a 1:1.8 ratio of PCR product to AMPure XP reagent to wash out fragments < 100 bp. The two purification methods produced similar results, and the cleaned amplicon DNA was used as template for the subsequent barcode PCR. To incorporate barcodes, 10 μl PCRs were carried out containing 2 μl template, 0.2 μmol of each forward and reverse barcode primer per microsatellite locus, and 5 μl MyTaq Red Mix. The thermocycling profile for all barcode PCRs consisted of 90 s of initial denaturation at 94°C, 8 cycles of 30 s at 94°C, 30 s at 60°C, 30 s at 72°C, and a final extension for 5 minutes at 72°C.

Products from the barcode PCR were purified either by gel purification or bead cleaning with AMPure XP reagent. For the gel purification method, the PCR products were run on 1.3% agarose and bands corresponding to expected product size were cut out, then amplicon DNA was isolated using the Qiagen gel purification kit. The cleaned products were quantified using a Qubit® Fluorometer and Qubit® dsDNA HS Assay Kit; both purification methods produced similar results and yielded 20–340 ng DNA. The samples, including negative controls, were pooled into libraries containing approximately equal amounts of amplicons from each individual and locus.

The libraries were prepared for sequencing and Illumina TruSeq adapters were incorporated using the KAPA Hyper Prep Kit. Amplicon libraries were sequenced on an Illumina MiSeq platform with V3 chemistry and 600 cycles to obtain at least 2,000 reader per locus per individual. Raw reads from the amplicon libraries were trimmed using BBDuk in Geneious with a minimum quality of 30. This step also removed Illumina adapters and discarded reads < 50 bp, as the expected microsatellite-containing amplicons were > 100 bp. The trimmed reads were paired by name and merged, then sequences in each library were separated by barcode in Geneious and a fastq file was created for each individual (adult or egg mass).

**Supplementary Table 1** PCR primer sequences designed to amplify microsatellite loci in *S. gigas.* Each forward and reverse primer was adapted on the 5’ end with a universal primer sequence from Vartia et al. (2016).

| **Locus** | **Forward primer (5’🡪3’)** | **Forward adapter** | **Reverse primer (5’🡪3’)** | **Reverse adapter** |
| --- | --- | --- | --- | --- |
| MS 03 | TGAACATGGGTCAGTCGGTC | M13 | ACCAAATCTCCGTGTCCAGG | CAG |
| MS 26 | ATGAGCCCAATGTCAACAGA | M13 | CCTCTCCCTTTTCCTCTTCCC | CAG |
| MS 31 | TTAACGAACCCCGGTTATCG | M13 | ACACGTAAATCATTGAAAGAGAGTG | CAG |
| MS 34 | TTTAGGTGGGGTGGGGTCTG | Neo | TTCTCTACGGAATGTGCCCC | CAG |

**Supplementary Table 2** Polymerase chain reaction (PCR) cycle conditions. The first PCR step (PCR 1) utilized locus-specific primers to amplify microsatellites in *S. gigas* adults and embryos. The second (PCR 2) incorporated combinatorial barcodes into the products from PCR 1 in order to tag individual limpets and embryo samples prior to sequencing. The same PCR 2 cycle was used for all PCR 1 products and barcodes.

| **PCR step** | **Locus/multiplex** | **Cycle** |
| --- | --- | --- |
| PCR 1 | MS-03 | 1. 94℃ for 1:30 2. **Denature:** 94℃ for 0:20 3. **Anneal:** 59℃ for 0:20 4. **Extend:** 72℃ for 0:25 5. Repeat steps 2-4 for 24 additional cycles 6. 72℃ for 10:00 7. Hold at 10℃ |
| PCR 1 | MS-26, MS-31, and MS-34 multiplex | 1. 94℃ for 1:30 2. **Denature:** 94℃ for 0:20 3. **Anneal:** 56℃ for 0:20 4. **Extend:** 72℃ for 0:25 5. Repeat steps 2-4 for 29 additional cycles 6. 72℃ for 10:00 7. Hold at 10℃ |
| PCR 2 | All | 1. 95℃ for 1:30 2. **Denature:** 95℃ for 0:30 3. **Anneal:** 60℃ for 0:30 4. **Extend:** 72℃ for 0:30 5. Repeat steps 2-4 for 7 additional cycles 6. 72℃ for 5:00 7. Hold at 10℃ |

**References**

Toonen RJ, Puritz JB, Forsman ZH, Whitney JL, Fernandez-Silva I, Andrews KR, Bird CE (2013) ezRAD: a simplified method for genomic genotyping in non-model organisms. PeerJ 1:e203. doi: 10.7717/peerj.203

Vartia S, Villanueva-Cañas JL, Finarelli J, Farrell ED, Collins PC, Hughes GM, Carlsson JEL, Gauthier DT, McGinnity P, Cross TF, FitzGerald RD, Mirimin L, Crispie F, Cotter PD, Carlsson J (2016) A novel method of microsatellite genotyping-by-sequencing using individual combinatorial barcoding. R Soc Open Sci 3:150565. doi: 10.1098/rsos.150565
